# Surfactant Protein (SP)-A Suppresses Preterm Delivery and Inflammation via TLR2

**DOI:** 10.1371/journal.pone.0063990

**Published:** 2013-05-20

**Authors:** Varkha Agrawal, Keith Smart, Tamas Jilling, Emmet Hirsch

**Affiliations:** 1 Department of Obstetrics and Gynecology, NorthShore University HealthSystem, Evanston, Illinois, United States of America; 2 Department of Obstetrics and Gynecology, Pritzker School of Medicine, University of Chicago, Chicago, Illinois, United States of America; 3 Department of Pediatrics, NorthShore University HealthSystem, Evanston, Illinois, United States of America; Charite Universitätsmedizin Berlin, Germany

## Abstract

Toll like receptors (TLRs) are pattern-recognition molecules that initiate the innate immune response to pathogens. Pulmonary surfactant protein (SP)-A is an endogenously produced ligand for TLR2 and TLR4. SP-A has been proposed as a fetally produced signal for the onset of parturition in the mouse. We examined the effect of interactions between SP-A and the pathogenic TLR agonists lipopolysaccharide (LPS), peptidoglycan (PGN) and polyinosinic:cytidylic acid (poly(I:C)) (ligands for TLR4, TLR2 and TLR3, respectively) on the expression of inflammatory mediators and preterm delivery. Three types of mouse macrophages (the cell line RAW 264.7, and fresh amniotic fluid and peritoneal macrophages, including macrophages from TLR4 and TLR2 knockout mice) were treated for up to 7 hours with pathogenic TLR agonists with or without SP-A. SP-A alone had no effect upon inflammatory mediators in mouse macrophages and did not independently induce preterm labor. SP-A significantly suppressed TLR ligand-induced expression of inflammatory mediators (interleukin (IL)-1β, tumor necrosis factor (TNF)-α and the chemokine CCL5) via a TLR2 dependent mechanism. In a mouse inflammation-induced preterm delivery model, intrauterine administration of SP-A significantly inhibited preterm delivery, suppressed the expression of proinflammatory mediators and enhanced the expression of the CXCL1 and anti-inflammatory mediator IL-10. We conclude that SP-A acts via TLR2 to suppress TLR ligand-induced preterm delivery and inflammatory responses.

## Introduction

Preterm birth is the most important cause of neonatal morbidity and mortality not due to congenital anomalies in the developed world [1]. Up to 40% of cases of preterm labor are associated with infection in the gestational compartment [2]. Although in individual cases it may be difficult to determine whether infection is a cause or a consequence of labor, it has become clear that infection and inflammation represent important and frequent mechanisms of disease.

Toll like receptors (TLRs) are a family of membrane bound proteins that recognize pathogen-associated molecular patterns and mediate innate immune responses [3]–[6]. Binding of TLRs is the initial event in activation of the innate immune system which leads, among other events, to the nuclear translocation of the transcription factor nuclear factor (NF)-κB and the elaboration of a network of inflammatory mediators. Our lab and others have shown that preterm labor can be induced in mice by pathogens or pathogen-derived TLR ligands for TLR4 (which recognizes Gram negative bacteria) [7]–[9], TLR2 (which recognizes Gram positive bacteria) [10], TLR3 (which recognizes viral intermediates) [11], and in a synergistic fashion, TLR2 plus TLR3 [12].

In addition to exogenous (microbial) ligands, TLRs can bind substances produced by the host (‘endogenous ligands’). Surfactant protein (SP)-A, an endogenous ligand for TLR2 [13]–[15] and TLR4 [16], is of particular relevance to human gestation. SP-A is synthesized by the fetal lung starting in the 28^th^ week of human pregnancy, reaching fully functional levels at about the 34^th^ week [17], [18]. In addition to the lung, SP-A is expressed in other cells and tissues [19], including those of the female reproductive tract [19]–[23]. The production of surfactant is the major determinant of fetal lung maturity, which in turn is the major factor determining *ex utero* survival of the fetus. It has been suggested that in mice, the appearance of fetal SP-A toward the end of gestation acts as an intrauterine signal for the onset of parturition via an inflammatory mechanism [24].

SP-A is a member of the collectin family of proteins. Collectins have a C-terminal globular region with a carbohydrate recognition domain (CRD, or lectin domain), a hydrophobic alpha-helical neck region, a collagen-like region with Gly-X-Y repeats and an N-terminal disulfide bond-forming region [25]. SP-A may act either as a pro-inflammatory or anti-inflammatory agent, depending on a variety of circumstances [25], including the type of receptor engaged [26]. In the absence of a pathogen, SP-A binds through its lectin domain to signal-inhibitory regulatory protein-α (SIRP-α). In the presence of a foreign organism or cellular debris, to which the lectin domain of SP-A binds, the free collagen-like region activates immune cells through the CD91-calreticulin complex. Engagement of the different receptors elicits different responses. When SP-A binds SIRP-α, inflammatory-mediator production is inhibited. By contrast, SP-A enhances inflammatory mediator production through the CD91-calreticulin complex.

Using a well-validated mouse model of infection-induced preterm delivery, we demonstrated previously that combined activation of TLR2 and TLR3 using peptidoglycan (PGN) and polyinosinic:cytidylic acid (poly(I:C)) yields dramatic synergy in the labor response and uterine expression of inflammatory mediators [12]. We hypothesized that infection-induced labor might involve a ‘two-hit’ trigger mechanism that protects the pregnancy from an overactive response to minor infection while providing an amplification mechanism for more serious or combined infections that threaten the well-being of the mother and/or fetus. Given the reported proinflammatory role of the endogenous TLR ligand SP-A in signaling for mouse parturition, here we examine the possibility that SP-A may serve as one of these two ‘hits’. We test the effects of SP-A on the labor- and inflammation-inducing actions of pathogen-derived TLR ligands. We find that, when administered into the uterine lumen outside of the fetal membranes and in contrast to our expectations, SP-A suppresses labor and inflammation, rather than inducing them.

## Materials and Methods

### Ethics Statement

All procedures involving animals were approved by the NorthShore University HealthSystem Animal Care and Use Committee and conform to the Guide for Care and Use of Laboratory Animals (1996, National Academy of Sciences).

### Cell Culture

Three sources of macrophages were used for cell culture experiments: 1) the mouse macrophage cell line RAW 264.7 (American Type Culture Collection TIB-71); 2) thioglycolate-stimulated peritoneal macrophages from male mice of the following mouse strains (all purchased from the Jackson Laboratory, Bar Harbor, ME): B6.B10ScN-Tlr4lps-del/JthJ (TLR4-KO), B6.129-Tlr2tm1Kir/J (TLR2-KO), C57BL/6J (wild-type (WT) controls), C3H/HeJ (natural hyporesponsive TLR-4 mutant) and C3HeB/FeJ (WT control); and 3) amniotic fluid macrophages from the CD-1 mice on day 14.5 and 16.5 of pregnancy.

RAW 264.7 cells were cultured in DMEM High Glucose (GIBCO 11965-092) supplemented with 10% fetal bovine serum, 1% streptomycin and 1% penicillin in tissue culture flasks at 37°C in 5% CO_2_/95% air and were passaged every 2 or 3 days to maintain logarithmic growth. Prior to each experiment, cells (4×10^5^ cells per well) were plated in triplicate in 6-well plates and cultured overnight.

Peritoneal macrophages were freshly harvested as previously described [27]. Briefly, mice were injected intraperitoneally (i.p.) with 1 ml of 3% thioglycolate. Three days after injection, mice were euthanized by CO_2_ inhalation and peritoneal exudate cells were isolated by flushing the peritoneal cavity with 15 ml ice-cold PBS. These cells were centrifuged, washed once, plated at a density of 1.5×10^6^ cells per well and incubated for 2 hours in 6-well plates under similar conditions as described above, supplemented with 10 mM HEPES. Adherent cells were confirmed as macrophages using the F4/80 macrophage cell marker.

Amniotic fluid macrophages were harvested from pregnant CD-1 mice on days 14.5 or 16.5 of pregnancy. Amniotic fluid was aspirated from amniotic sacs using an 18-G needle, mixed in RPMI 1640 medium (ATCC 30-2001) in 12-well plates and incubated for 1 hour. Medium was changed and adherent cells were used for experiments. In separate incubations, these adherent cells were confirmed as macrophages using the F4/80 antibody macrophage cell marker.

Cells were cultured for up to 7 hours with either LPS (TLR4 agonist, 5 ng/ml), PGN (TLR2 agonist, 1 µg/ml), poly(I:C) (TLR3 agonist, 10 µg/ml), PGN plus poly(I:C), or medium. The effect of either simultaneous or sequential incubation with SP-A (20 µg/ml) was studied (i.e. SP-A added either together with, before or after TLR ligands, with an intermediate washing step). All *in-vitro* experiments were conducted in triplicate and repeated twice (i.e. three triplicate experiments).

Viability of cultured cells was assessed using trypan blue dye exclusion. For RAW 264.7 cells, viability prior to plating was 95% and 5 hours after plating was as follows for each treatment group: 93.5% for control (medium) treatment; 90% for LPS - 91.3% for PGN; 92.5% for poly(I:C); 90.5% for PGN+poly(I:C); 90% for SP-A; 90.5% for SP-A+LPS; 88% for SP-A+ PGN+poly(I:C). For peritoneal macrophages, the corresponding viability values were: pre-plating: 92%; after plating: control - 90%; LPS −88.4%; PGN −87%; poly(I:C) –88.1%; PGN plus poly(I:C) −85%; SP-A −89.5%; SP-A+LPS –87.3%; SP-A+ PGN+poly(I:C) –83.21%. For amniotic fluid macrophages, the corresponding viability values were: after plating: control-87.69; LPS −84.8%. The differences between post-plating values were not statistically significant from each other.

### SP-A Purification

SP-A purification was done from bronchial lavage fluid collected from patients with alveolar proteinosis. The lavage fluid was donated by and was processed according to method reported by Condon [24]. Briefly, lavage fluid was delipidated by centrifugation at 10,000 rpm for 10 minutes at 4°C. The supernatant was discarded and a mix of isopropyl ether/1-butanol/RNAse free water was added. The solution was vortexed vigorously every 5 minutes for 30 minutes and frozen overnight at −80°C, then centrifuged at 2500 rpm for 10 minutes. The upper organic layer was discarded. The protein was ethanol-precipitated from the aqueous layer and suspended in cold RNAse-free water. Serum albumin was removed by extraction on a DEAE-Affigel blue column (Bio-Rad). Endotoxin decontamination was done using polymyxin B agarose (Sigma) at 37°C for 30 minutes. The endotoxin content in SP-A was ∼0.3 pg/µg of protein as determined by the QCL-1000 Limulus amebocyte lysate assay (Lonza, Walkersville, MD). Purity of the delipidated, DEAE-Affigel blue- and polymyxin B-treated SP-A preparation was assessed by Coomassie blue staining and western blot analysis using an SP-A-specific antibody. Protein concentration was checked by BCA protein assay (Thermo scientific, Rockford, IL).

### RT-PCR Analysis

At the end of cell culture experiments, medium was aspirated and cells were washed twice with PBS and then lysed in the wells with TRIzol reagent (Invitrogen, Carlsbad, CA) to extract total RNA according to the manufacturer’s protocol. For tissue specimens, total RNA was extracted after homogenization in TRIzol reagent.

The quantity and quality of the RNA were verified by spectrophotometry and formaldehyde gel electrophoresis, respectively. Two µg of total RNA were used as a template for cDNA synthesis. cDNA was prepared using random primers and the Moloney Murine Leukemia Virus (MMLV) reverse transcriptase system (Invitrogen). All PCR primers and probes were purchased from Applied Biosystems (Foster City, CA) (IL-1β Mm00434228; CCL5 (RANTES) Mm01302428; TNF Mm00443258; CXCL1 Mm00433859; IL-10 Mm00439614; Mouse glyceraldehyde 3-phosphate dehydrogenase (GAPDH) (20X) 4452339E). Use of TaqMan PCR Reagent Kits was in accordance with the manufacturer's manual. Reactions were performed in a 10 µL mixture containing 0.5 µL cDNA. Duplex RT-PCR was performed with one primer pair amplifying the gene of interest and the other an internal reference (GAPDH) in the same tube. Thermocycler parameters were 50°C for 2 minutes, 95°C for 10 minutes, followed by 40 cycles of 95°C for 15 seconds, and 60°C for 1 minute. Semi-quantitative analysis of gene expression was done using the comparative CT (ΔΔCT) method, normalizing expression of the gene of interest to GAPDH. PGN/poly(I:C) values were set to 100. PCR assays were performed in duplicate for each of the triplicate cell culture samples.

### Antibodies

Primary antibodies were as follows: phosphorylated ERK (pERK, sc-7383, Santa Cruz Biotechnology, Santa Cruz, CA) and total ERK (sc-135900, Santa Cruz), IκBα (4814, Cell Signaling Technology, Danvers, MA), actin (AAN01, Cytoskeleton Inc., Denver, CO), F4/80 (ab6640, Abcam, Cambridge, MA) and IL-1β (ab9722, Abcam). Secondary antibodies were: goat anti-mouse IgG-HRP (sc-2031, Santa Cruz for pERK, ERK and IκBα), goat anti-rabbit IgG-HRP (sc-2030, Santa Cruz, for actin), rabbit anti-rat IgG-FITC (ab6730, Abcam for F4/80) and goat anti-rabbit IgG-FITC (sc-2012, Santa Cruz, for IL-1β).

### Protein Extraction and Western Blot Analysis

For western blotting from cell culture, cells were washed twice with cold PBS and lysed in ice-cold 1X radioimmune precipitation assay (RIPA) buffer (1X TBS, 1% Nonidet P-40, 0.5% Na deoxycholate, 0.1% SDS, 0.004% Na azide, protease inhibitor mixture set (PMSF, Na orthovanadate and protease inhibitor cocktail) and phosphatase inhibitors I and II (Santa Cruz)). Lysates were collected and incubated on ice for 1 hour and then centrifuged at 10,000 *g* for 10 minutes at 4°C. Supernatants were collected and used as whole cell lysates for western blotting. Protein concentration was measured spectrophotometrically (Nanodrop 2000, Thermo Scientific) at A_280_. Equal amounts of protein (50 µg) from cell lysates were separated by SDS-PAGE and blotted onto PVDF transfer membranes. The membranes were blocked either at room temperature for 1 hour or at 4°C overnight. 5% BSA or 5% nonfat dry milk in TBS-T buffer (10 mM Tris, 0.1 M NaCl, 0.1% Tween 20, pH 7.4) was used as blocking buffer for the detection of phosphorylated and non-phosphorylated proteins, respectively. Blots were incubated with the appropriate primary and secondary antibodies either for 1–2 hour at room temperature or overnight at 4°C with slight agitation. Proteins were detected using the ECL western blot detection system (GE Healthcare Bio-Sciences Corp., Piscataway, NJ) in a Storm imager (Molecular Dynamics, San Diego, CA).

After detection of signal for pERK, membranes were stripped by incubating in stripping buffer (20 Mm Glycine, 0.1% SDS, 1% Tween 20, pH 2.2) at 50°C for 30–40 minutes. Following stripping, blots were washed five times for 10 minutes each with TBST buffer, redeveloped to check for the removal of pERK signal, and re-probed with primary antibody for total ERK. IκBα was detected on a separate membrane with actin as a loading control. Quantification of bands was performed using ImageQuant software (Molecular Dynamics). Background intensity was subtracted from each sample and then fold change was determined (phosphor-proteins/total protein or target protein/loading control). Control values were set to 100. Each experiment was done twice in duplicate.

### NF-κB Reporter Gene Assay

RAW-Blue™ (InvivoGen, San Diego, CA) is a mouse macrophage reporter cell line that stably expresses a secreted embryonic alkaline phosphatase (SEAP) gene inducible by the transcription factor NF-κB. RAW-Blue cells were cultured under similar conditions as mentioned above, in medium supplemented with Zeocin, a selection marker (200 µg/ml). Prior to each experiment, cells (4×10^5^ cells per well) were plated in triplicate in 12-well plates, cultured overnight and stimulated with either LPS (5 ng/ml) or PGN plus poly(I:C) (2 µg/ml and 20 µg/ml, respectively) for 4 hours in the presence or absence of SP-A (20 µg/ml). Supernatants were incubated with a detection substrate (QUANTI-Blue™, InvivoGen) at 37°C for 30 minutes, and relative SEAP activity was determined using a spectrophotometer at 620–655 nm.

### Immunofluorescence

At the end of cell culture experiments, medium was aspirated and cells were washed twice with PBS and fixed in 4% formaldehyde. Cells were blocked and permeabilized with 3% BSA in PBS with 0.1% Triton X-100, and incubated overnight with primary antibody for F4/80 or IL-1β in 1% BSA in PBS at 4°C. Cells were then incubated with the appropriate secondary antibody labeled with FITC for 45 min at room temperature. To visualize the nuclei cells were fixed in Prolong gold antifade reagent with DAPI (Invitrogen). Fluorescent antigen distribution was examined with a Leica DMLB florescence microscope (Wetzlar, Germany), and images were captured using a Retina 1350B camera (Q Imaging, Canada) and IPlab image acquisition/analysis software (Scanalytics/BD Biosciences, Rockville, MD).

### Mice

For pregnancy outcome experiments, CD-1 female mice in estrus were selected by the gross appearance of the vaginal epithelium and were impregnated naturally. Mating was confirmed by the presence of a vaginal plug. Intrauterine (IU) injections were performed on day 14.5 of a 19–20 day gestation, as previously described [28]. Briefly, animals were anesthetized with 0.015 ml/g body weight of Avertin (2.5% tribromoethyl alcohol and 2.5% tert-amyl alcohol in PBS). A 1.5 cm midline incision was made in the lower abdomen. In the mouse, the uterus is a bicornuate structure in which the fetuses are arranged in a ‘beads-on-a-string’ pattern. Mice underwent injection of either (a) LPS (0.025 mg/mouse); (b) PGN (0.3 mg/mouse) plus poly(I:C) (1.0 mg/mouse); or (c) saline. PGN and poly(I:C) were combined because we showed previously that this results in synergistic effects (both preterm delivery and inflammatory responses), a phenomenon that is mediated by both the MyD88-dependent and the MyD88-independent signaling pathways downstream of the TLR receptors [12]. Each of the above intrauterine injections was performed in the midsection of the right uterine horn at a site between two adjacent fetuses, taking care not to inject individual fetal sacs, and in the presence or absence of SP-A (0.075 or 0.105 mg/mouse). Surgical procedures lasted approximately 10 minutes. The abdomen was closed in two layers, with 4–0 polyglactin sutures at the peritoneum and wound clips at the skin.

Animals recovered in individual, clean cages in the animal facility. Observations were made twice-daily for both preterm delivery and maternal health status. Preterm delivery was defined as the finding of at least one fetus in the cage or in the lower vagina within 48 hours of surgery. Necropsies were performed either after delivery or at the latest by 48 hours after surgery. The number of fetuses delivered or remaining *in utero* and the survival status of these retained fetuses (as determined by cardiac or vascular pulsations in the fetal bodies or membranes) were recorded.

### Tissue Harvest

For experiments requiring tissue harvests, animals were euthanized 8 hours after surgery. The inoculated horn was incised longitudinally along the anti-mesenteric border. Gestational tissues (uteri (full thickness biopsies from the middle region), fetal membranes (pooled from all conceptuses in the injected uterine horn), fetuses and placentas) were harvested, washed in ice-cold PBS, flash-frozen in liquid nitrogen and stored at −85°C for later RNA extraction.

### Statistical Analysis

Continuous variables (e.g. relative mRNA levels) were assessed with Student’s t-test or ANOVA or, when data were not normally distributed and two groups were compared, the Mann-Whitney U test. Categorical variables (e.g. preterm delivery) were analyzed using Fisher’s Exact Test. *P*<0.05 was considered a statistically significant difference.

## Results

### SP-A Suppresses TLR Ligand-induced Expression of Inflammatory Mediators

**Figure 1:**
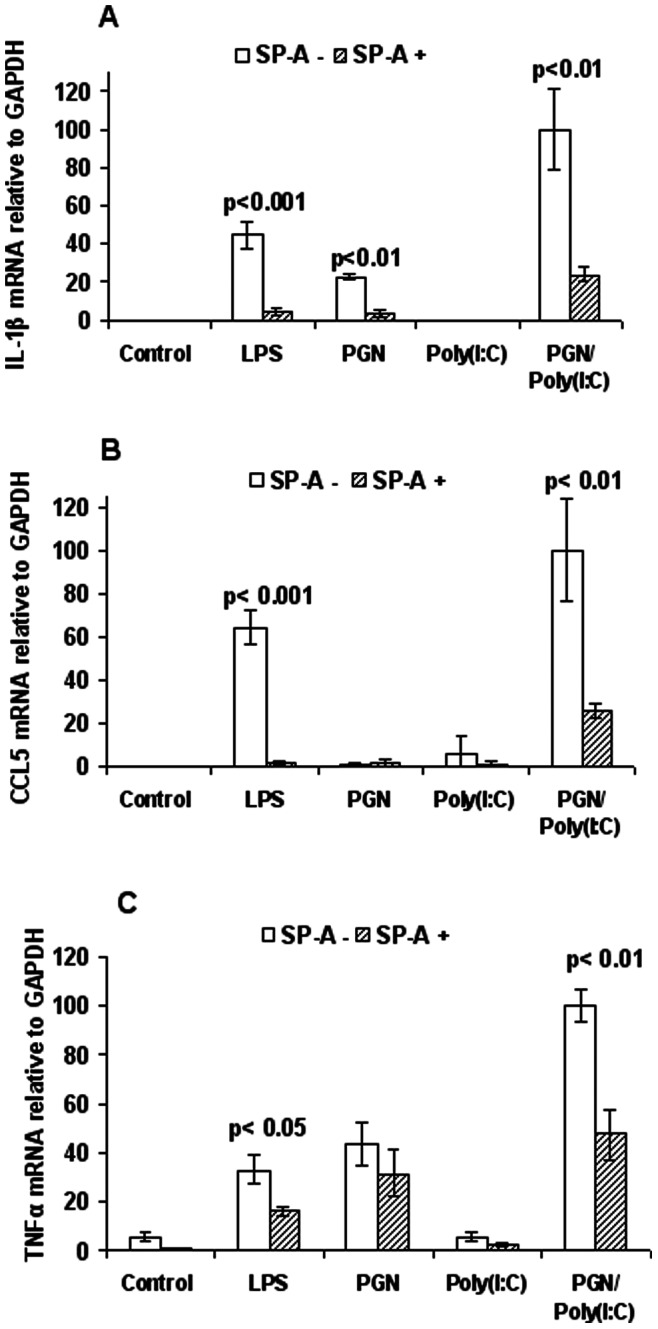
SP-A suppresses TLR ligand-induced expression of inflammatory mediators. Expression by RT-PCR of IL-1β (A), CCL5 (B) and TNF-α (C) in the RAW 264.7 macrophage cell line 5 hours after treatment with PBS, LPS (a TLR4 ligand), PGN (a TLR2 ligand), poly (I:C) (a TLR3 ligand) and PGN+poly(I:C), with or without SP-A. Concentrations of reagents are provided in the text (Methods). P values were calculated by t-test and compare exposures with and without SP-A**.** n = 3 replicates per condition per experiment. Depicted is a representative figure from three repeat experiments. Error bars = standard deviation. Values for PGN+Poly(I:C) group were set to 100.

To study inflammatory responses in one of the key cell types implicated in infection-induced labor, we used an *in vitro* macrophage culture system (Figure 1). Toll-like receptor signaling was induced by either LPS (a TLR4 ligand) or the combination of PGN+poly(I:C) (TLR2 and TLR3 ligands, respectively). These two approaches are alternative methods of simultaneously activating both of the alternative intracellular signaling pathways for TLRs, the myeloid differentiation primary-response gene 88 (MyD88)-dependent and the MyD88-independent pathways. TLR activation in RAW264.7 macrophages induces a characteristic expression pattern of mRNAs for interleukin (IL)-1β, the chemokine CCL5 and tumor necrosis factor (TNF)-α. These inflammatory markers were chosen to represent activation of the MyD88-dependent pathway, the MyD88-independent pathway and both pathways, respectively, and because mRNA levels for these genes are known to correlate closely with active protein [29]. A previously described synergistic response to TLR2+TLR3 [12] was confirmed in the present results. SP-A significantly suppresses inflammatory responses to activation of TLR4 and TLR2+TLR3, with partial suppression of the response to activation of TLR2 alone. SP-A alone had no effect upon inflammatory mediators. The anti-inflammatory cytokine IL-10 was not detectable in any group.

### The Anti-inflammatory Effect of SP-A does not Depend Upon Direct Interaction with the Stimulating Ligand

**Figure 2:**
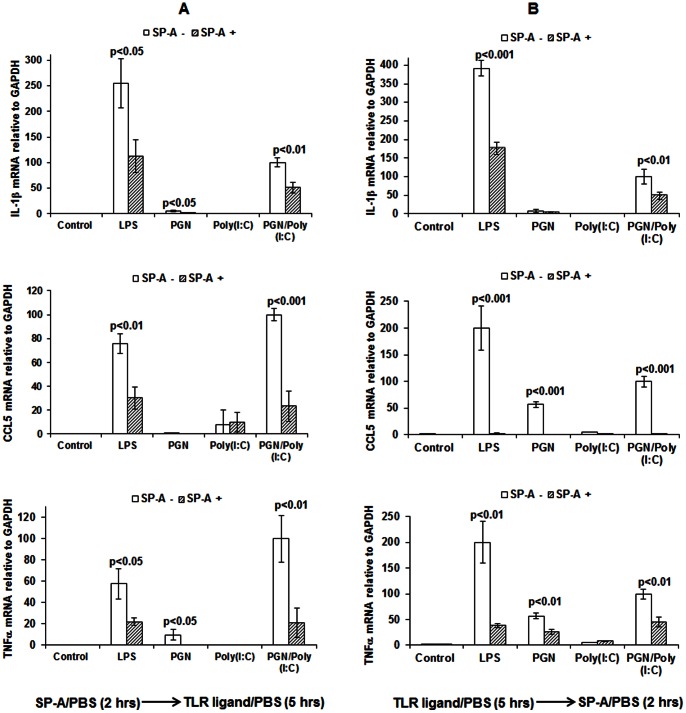
The anti-inflammatory effect of SP-A does not depend upon direct interaction with the stimulating ligand. Expression by RT-PCR of IL-1β, CCL5 and TNF-α in RAW 264.7 cells after sequential treatment with either SP-A followed by TLR ligand (Panel A) or TLR ligand followed by SP-A (Panel B). (A) initial incubation for 2 hours with PBS or SP-A followed by washing and incubation for an additional 5 hours with PBS, LPS, PGN, poly (I:C) or PGN+poly(I:C).(B) initial incubation for 5 hours with PBS, LPS, PGN, poly (I:C) or PGN+poly(I:C), followed by washing and incubation for an additional 2 hours with either PBS or SP-A. P values were calculated by t-test and compare exposures with and without SP-A. n = 3 replicates per condition per experiment. Depicted is a representative figure from three repeat experiments. Error bars = standard deviation. Values for PGN+Poly(I:C) group were set to 100.

To investigate the possibility that inhibition by SP-A of TLR ligand-induced inflammation depends upon direct physical interaction between these two molecules (i.e. sequestration or similar mechanism), we performed sequential incubations in RAW 264.7 cells, with SP-A incubations occurring either before or after incubation with TLR ligands. In all cases, an intermediate washing step (3 times with phosphate-buffered saline (PBS)) was performed between the first and second exposure (Figure 2). SP-A suppressed TLR ligand-induced expression of IL-1β, CCL5 and TNF-α regardless of the sequence of exposure, suggesting that this phenomenon is cell-dependent and is not due to sequestration of the TLR ligand by SP-A within the medium.

### The Anti-inflammatory Effect of SP-A is Receptor-mediated and Requires TLR2

**Figure 3:**
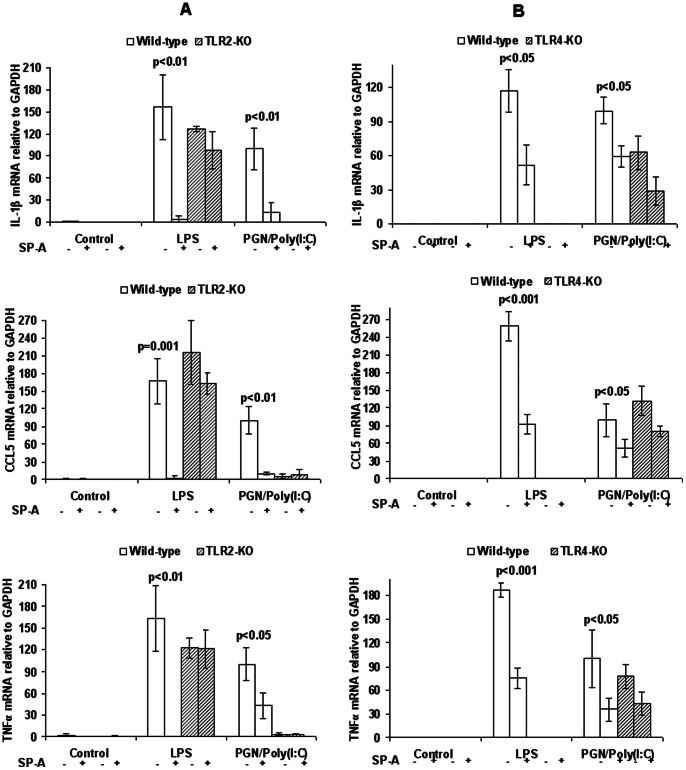
The anti-inflammatory effect of SP-A requires TLR2. Expression by RT-PCR of IL-1β, CCL5 and TNF-α in WT and (Panel A) TLR2-KO and (Panel B) TLR4-KO peritoneal macrophages after treatment with PBS, LPS, or PGN+poly(I:C), with or without SP-A. P values were calculated by ANOVA and compare four treatment groups (KO or WT with or without SP-A) for each TLR ligand**.** n = 3 replicates per condition per experiment. Depicted is a representative figure from three repeat experiments. Error bars = standard deviation. Values for the PGN+Poly(I:C) group were set to 100.

To test whether the anti-inflammatory effect of SP-A is TLR-mediated, we used peritoneal macrophages isolated from TLR2-deficient (KO), TLR4-KO and WT control mice (Figure 3). Adherent cells from plated peritoneal exudates were confirmed as macrophages by staining for the cell marker F4/80 (Figure S1). Results similar to RAW 264.7 cells (shown in Figure 1, 2) were obtained with freshly isolated peritoneal macrophages from wild type mice. As expected, macrophages lacking TLR4 did not respond to LPS, and macrophages lacking TLR2 did not respond to PGN. This confirms the specificity of the exogenous ligands for their respective receptors and suggests that there was no significant contamination of reagents by endotoxin or other inflammatory stimulants. The anti-inflammatory effect of SP-A was eliminated in macrophages lacking TLR2 (Figure 3A) but was still present in macrophages lacking TLR4 (Figure 3B). Results similar to those for TLR4-KO macrophages were observed for macrophages extracted from C3H/HeJ mice, which carry a natural hyporesponsive mutation for TLR4 (Figure S2). Thus, the anti-inflammatory effect of SP-A is mediated at least in part by TLR2.

### SP-A Suppresses Activation of NF-κB

To identify intracellular mechanisms underlying suppression of the inflammatory response by SP-A, we focused on two signaling events downstream of TLR activation. The first is nuclear translocation of the transcription factor NF-κB, which occurs upon phosphorylation and subsequent degradation of its inhibitor IκBα (nuclear factor of kappa light polypeptide gene enhancer in B-cells inhibitor, alpha). Thus, degradation of IκBα can be used as an indirect measure of NF-κB activation. A second signaling event downstream of TLR activation is phosphorylation of extracellular signal-regulated kinases (ERK) -1 and ERK-2, mitogen-activated protein kinases that play important roles in cell proliferation, apoptosis, differentiation, cell migration, and gene expression.

**Figure 4:**
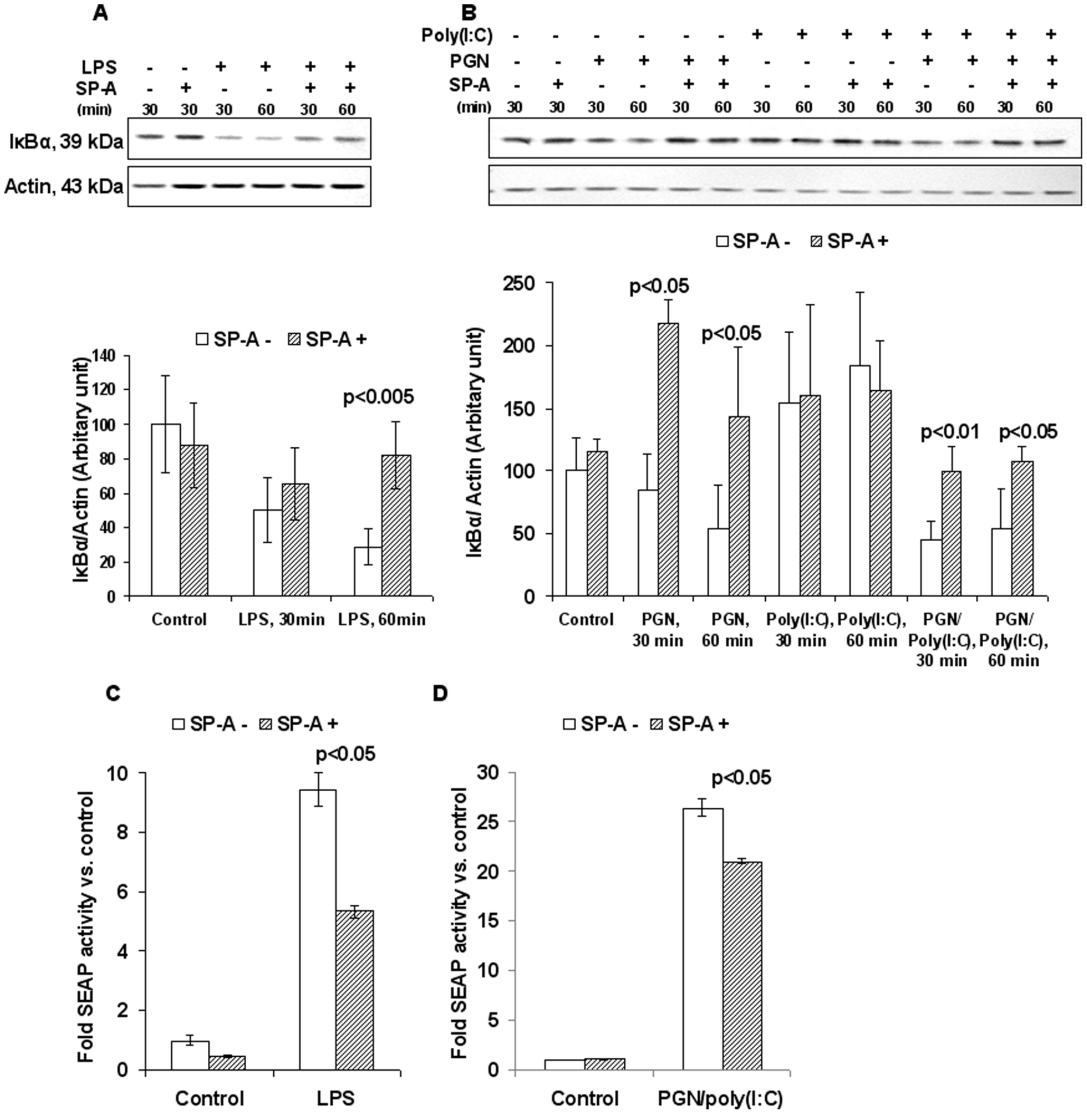
SP-A suppresses the NF-κB signaling pathway. *A* and *B*: Effect of SP-A on degradation of IκBα in macrophages stimulated with TLR ligands. Western blots (upper panels) and densitometric analysis (lower panels) of the effect of SP-A on levels of IκBα following treatment of RAW264.7 cells with either LPS (A) or PGN+poly(I:C) (B). Actin is used as a loading control. Representative blots of 2 independent experiments, each with duplicate sets, are shown. Values for control were set to 100 for densitometric analysis. *C* and *D*: SP-A attenuates TLR ligand-induced NF-κB activity in a mouse macrophage reporter cell line. NF-κB reporter gene assay in RAW-Blue™ cells showing the effect of SP-A on LPS- (C) and PGN+poly(I:C)- (D) induced NF-κB activity. Depicted is a representative figure from three repeat experiments. n = 3 replicates per condition per experiment. P values were calculated by t-test and compare exposures with and without SP-A. Error bars = standard deviation.

**Figure 5:**
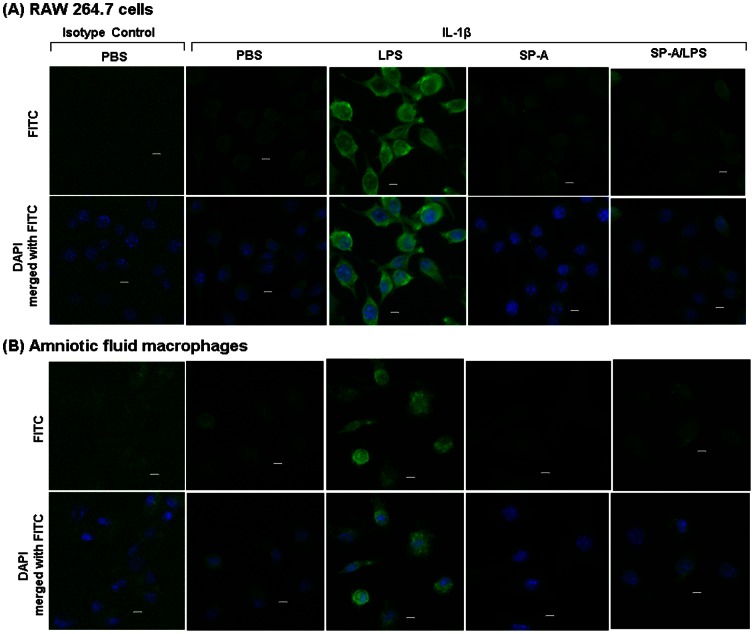
SP-A suppresses TLR ligand-induced expression of IL-1β in a macrophage cell line and in amniotic fluid macrophages. Immunocytochemistry with anti-IL-1β or an isotype-control antibody, each labeled with the fluorescent dye FITC, one hour after treatment with PBS or LPS with or without SP-A in RAW264.7 cells (A) and Day 16.5 amniotic fluid macrophages (B). n = 3 replicates per condition per experiment. Depicted is a representative figure from three repeat experiments. Top row: fluorescent microscopy for FITC (green). Bottom row: DAPI nuclear stain (blue), merged with FITC. Original magnification: 200X. Bars: 10 µm.

RAW 264.7 cells were cultured in the presence or absence of TLR ligands with or without supplemental SP-A. Degradation of IκBα resulting from treatment with LPS, PGN alone or PGN+poly(I:C) was reversed by SP-A (Figure 4A, 4B). SP-A alone had no effect upon IκBα. This observation was confirmed in a mouse macrophage cell line carrying an NF-κB reporter construct expressing secretory alkaline phosphatase (RAW-Blue™ cells) (Figure 4C, 4D). Furthermore, SP-A prevented the LPS-induced production of IL-1β protein, one of the main products of NF-κB activation, in cell culture (Figure 5A). SP-A had no effect on phosphorylation of ERK-1 and appeared to have an intermediate effect on the phosphorylation of ERK-2, not reaching statistical significance (Figure S3). Thus, SP-A has a suppressive effect on the NF-κB-mediated pro-inflammatory arm of TLR activation.

### Suppression of Inflammation by SP-A in Amniotic Fluid Macrophages

To address the possibility that the effects of SP-A might be compartment-specific (i.e. that macrophages within the amniotic sac might respond differently than those residing either within the uterus outside of the fetal membranes or within the peritoneal cavity), we isolated amniotic fluid macrophages from CD-1 mice on day 14.5 and 16.5 of pregnancy. Adherent cells were confirmed as macrophages using the specific F4/80 marker (Figure S4). IL-1β protein was not detectable by immunocytochemistry in day 14.5 amniotic fluid macrophages. SP-A abolished the LPS-induced expression of IL-1β protein in day-16.5 amniotic fluid macrophages (Figure 5B). SP-A alone had no effect upon IL-1β protein.

### Intrauterine SP-A Suppresses TLR Ligand-induced Preterm Delivery

In order to test whether the *in vitro* anti-inflammatory effect of SP-A has an *in vivo* correlate, a well-validated mouse model of infection-induced preterm labor using local administration of bacterial products was employed. The right uterine horns of pregnant mice on day 14.5 of a 19–20 day gestation were injected with TLR ligands (either LPS or PGN+poly(I:C)) with or without SP-A. These injections were performed in the space between the uterine lumen and the fetal sacs. Delivery of one or more pups within 48 hours was considered preterm. This model been shown to reproduce faithfully many aspects of infection-induced preterm labor in women, including the expression of cytokines, prostaglandins and other mediators; the lack of dependence on a drop in circulating progesterone; neonatal brain injury; and other characteristics [9], [28], [30], [31].

Neither sterile PBS nor SP-A alone produced preterm delivery in any of the animals tested. However, SP-A caused significant suppression in the rate of preterm delivery due to either LPS (38% vs. 90%, *p*<0.02) or PGN+poly(I:C) (18% vs. 73%, *p*<0.01) and led to higher rates of retention of fetuses *in utero* 48 hours after surgery (Table 1).

**Table 1:** Intrauterine SP-A suppresses TLR ligand-induced preterm delivery and leads to higher rates of retention of fetuses *in utero*.

Dose (mg/mouse)	N	Pretermdelivery (%)	Timing of delivery (hours)			Mean number of pupsdelivered within 48 h	Mean number of fetusesin-utero at 48 h
			<18	18–24	24–36		
PBS	5	0/5 (0)	0	0	0	0	12.4±1.14
SP-A (0.075 or 0.105)	5	0/5 (0)	0	0	0	0	10.2±2.58
LPS (0.025)	10	9/10 (90)	9	0	0	7.4±2.9	3.8±2.3
LPS+SP-A (0.075 )	13	5/13 (38)^a^	3	2	0	1.5±2.8^a^	10.0±3.7^a^
PGN (0.3)+poly(I:C) (1.0)	15	11/15 (73)	4	6	1	2.1±4.5	5.0±5.09
PGN+poly (I:C)+SP-A (0.075)	9	5/9 (55)	2	2	1	2.8±4.2	8.4±4.9
PGN+poly (I:C)+SP-A (0.105)	11	2/11 (18)^a^	0	2	0	0.5±1.7^a^	10.3±3.8^a^

Delivery of one or more pups within 48 hours was considered preterm. The number of pups per dam is shown as mean ± SD.

a P<0.05 compared to no SP-A treatment, Fisher’s exact test.

### SP-A Suppresses the Expression of Pro-inflammatory Mediators and Enhances the Expression of Anti-inflammatory Mediators in Gestational Tissues *in vivo*

**Figure 6:**
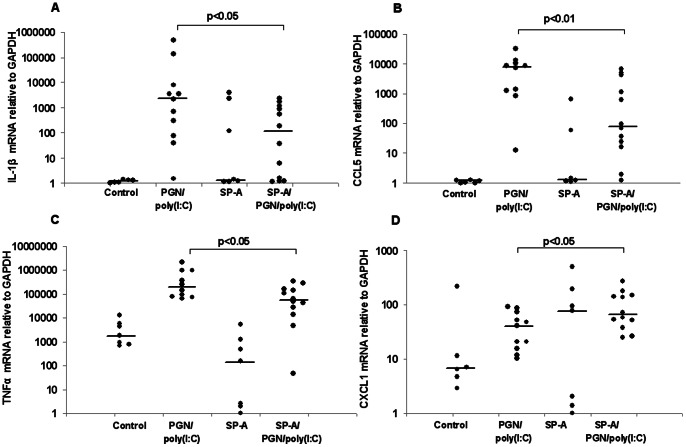
Effect of SP-A on the TLR ligand-induced expression of inflammation markers in the placenta. RT-PCR analysis of mRNA for IL-1β (A), CCL5 (B), TNF-α (C) and CXCL1 (D) normalized to GAPDH in placentas collected from CD-1 mice on day 14.5 of a 19- to 20-day gestation 8 hours after intrauterine administration of either PBS or PGN+poly(I:C) with or without SP-A. No transcripts were detected for IL-10. Data depicted are expression levels of cytokine for individual mice. N = 7 for control, n = 11 for PGN/poly(I:C), n = 7 for SP-A and n = 12 for SP-A/PGN/poly(I:C). Median values are depicted by a short straight line. P values were calculated by Mann-Whitney U test and compare PGN+poly(I:C) with and without SP-A.

**Figure 7:**
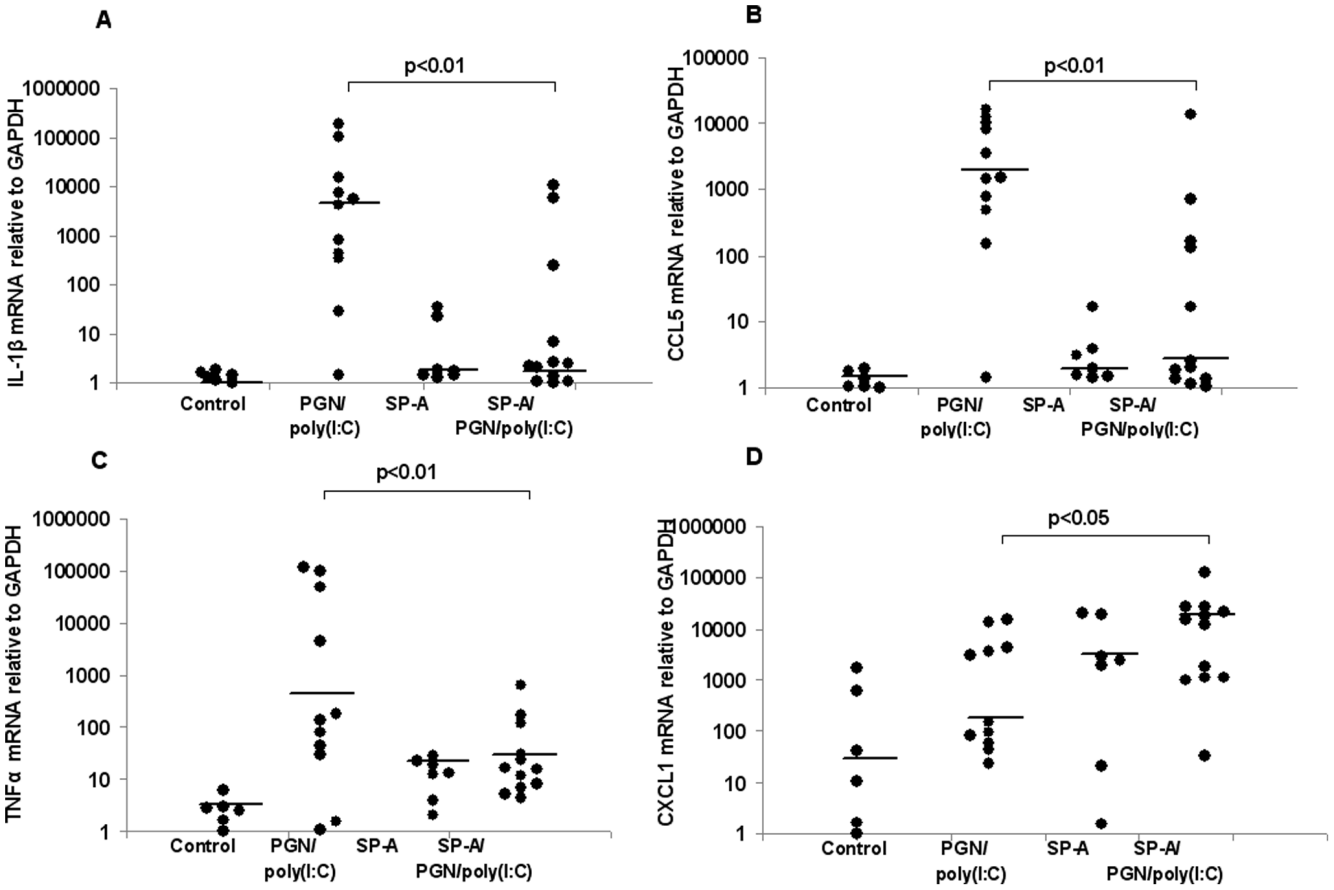
Effect of SP-A on the TLR ligand-induced expression of inflammation markers in fetal bodies. RT-PCR analysis of mRNA for IL-1β (A), CCL5 (B), TNF-α (C) and CXCL1 (D) normalized to GAPDH in fetal bodies collected from CD-1 mice on day 14.5 of a 19- to 20-day gestation 8 hours after intrauterine administration of either PBS or PGN+poly(I:C) with or without SP-A. No transcripts were detected for IL-10. Data depicted are expression levels of cytokine for individual mice. N = 6 for control, n = 11 for PGN/poly(I:C), n = 7 for SP-A and n = 12 for SP-A/PGN/poly(I:C). Median values are depicted by a short straight line. P values were calculated by Mann-Whitney U test and compare PGN+poly(I:C) with and without SP-A.

**Figure 8:**
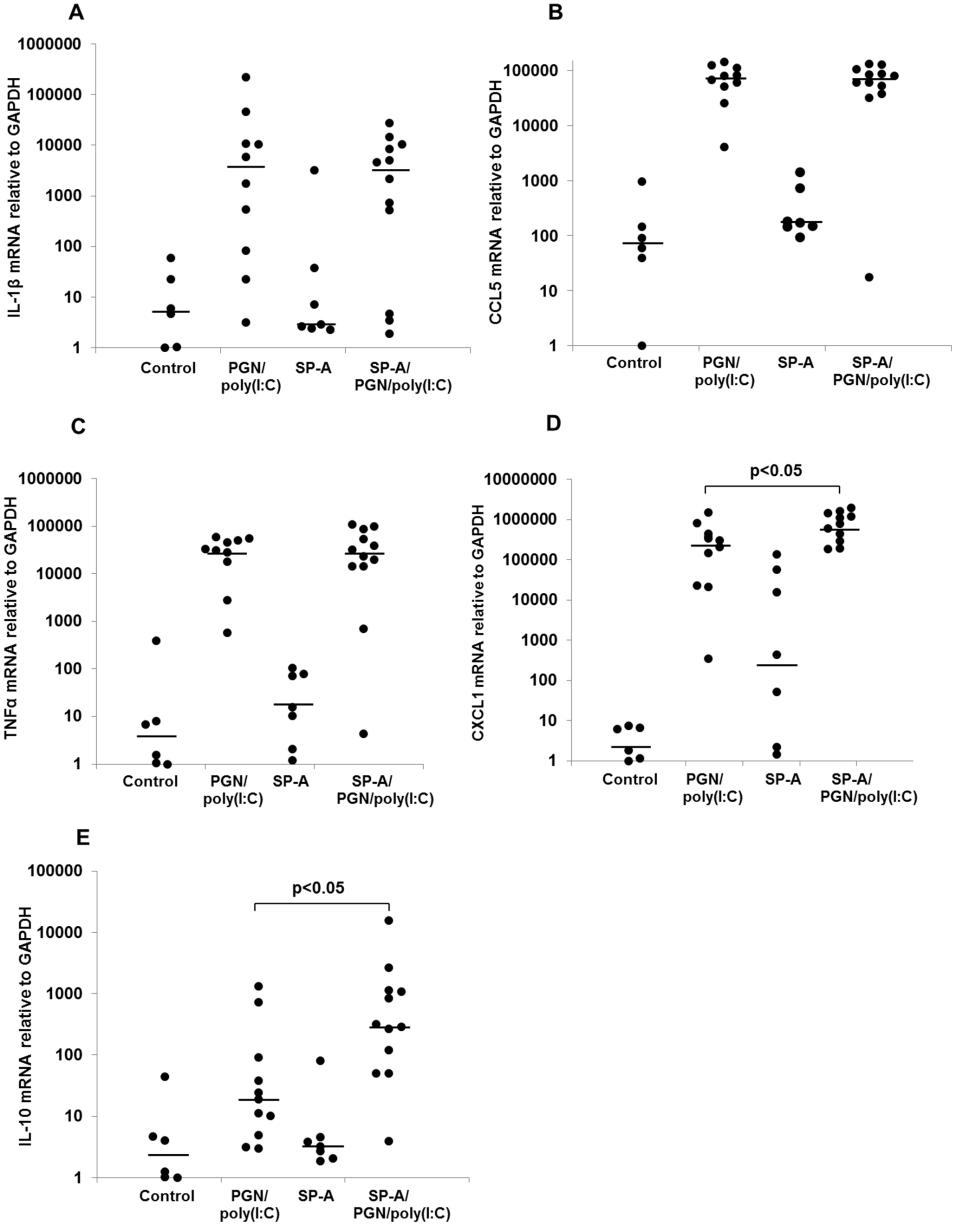
Effect of SP-A on the TLR ligand-induced expression of inflammation markers in uteri. RT-PCR analysis of mRNA for IL-1β (A), CCL5 (B), TNF-α (C), CXCL1 (D) and IL-10 (E) normalized to GAPDH in uteri collected from CD-1 mice on day 14.5 of a 19- to 20-day gestation 8 hours after intrauterine administration of either PBS or PGN+poly(I:C) with or without SP-A. Data depicted are expression levels of cytokine for individual mice. N = 6 for control, n = 11 for PGN/poly(I:C), n = 7 for SP-A and n = 12 for SP-A/PGN/poly(I:C). P values were calculated by Mann-Whitney U test compare PGN+poly(I:C) with and without SP-A.

Uteri, fetal membranes, fetuses and placentas were harvested 8 hours after intrauterine exposure to either PBS, SP-A, PGN+poly(I:C), or SP-A plus PGN+poly(I:C) and washed. Total RNA was extracted and real-time PCR was performed to determine the relative quantitative expression of CXCL1, the pro-inflammatory genes IL-1β, CCL5 and TNF-α and the anti-inflammatory gene IL-10 (Figure 6, 7, 8, Figure S5). IL-10 was detectable only in uteri, while the other transcripts were detectable in all gestational tissues examined. Treatment with PGN+poly(I:C) resulted in up-regulation of all these transcripts in all of the tissues in which they were detectable. SP-A significantly suppressed the expression of pro-inflammatory markers induced by PGN+poly(I:C) in placentas (Figure 6) and fetal bodies (Figure 7) but not in uteri (Figure 8) and fetal membranes (Figure S5). In a complementary fashion, SP-A significantly increased the expression of anti-inflammatory factors in placentas, fetal bodies and uteri. SP-A alone had no effect upon inflammatory mediators. No effect of SP-A was seen in fetal membranes.

## Discussion

A substantial body of evidence has accumulated showing that TLR signaling plays an important role in infection- and inflammation-induced preterm labor. In the present study we report that SP-A suppresses TLR ligand-induced preterm delivery and the expression of inflammatory mediators while increasing the expression of anti-inflammatory mediators *in vivo*. In macrophages (a key effector cell for infection-induced preterm labor), suppression of inflammation by SP-A is not due to sequestration of stimulating ligand by SP-A, is accompanied by decreased degradation of IκBα, and is TLR2 dependent.

Other studies also report that the mechanism for inhibition of PGN-induced cellular responses by SP-A involves interaction of SP-A with TLR2 [32]. In a macrophage cell line and alveolar macrophages, SP-A reduced zymosan-elicited NF-κB activation by altering zymosan-TLR2 interaction and down-regulating TLR2-mediated signaling and zymosan-stimulated TNFα secretion [13]. The mechanism for this suppression does not require binding of LPS by SP-A. It is also reported that SP-A recognizes a peptide component of CD14 and alters LPS-CD14 interaction [13], [15], [33].

An inhibitory effect of SP-A on the production of different inflammatory mediators has been reported in various *in vitro* and *in vivo* studies [15], [26], [34], [35]. Conversely, some studies have reported stimulatory [16], [36]–[38] or mixed [39] activity for SP-A. The underlying determinants of whether SP-A has pro-inflammatory or anti-inflammatory effects are not clear in all circumstances, but at least one mechanism involves the differential binding of alternative functional domains to specific receptors, as reviewed above (20, 21).

Our results stand in contradistinction to those of Condon [24], who reported that SP-A, which is produced by the fetal lung in late gestation and is important for *ex utero* survival, is an inflammatory signal for the onset of parturition. A potential role for SP-A in the extra-membranous space within the pregnant uterine lumen is unknown, as is the degree to which amniotic fluid SP-A crosses the fetal membranes or is otherwise transported to other compartments. The discrepant observations between the present *in vivo* study (in which SP-A was administered into the extra-amniotic space within the uterus) and that of Condon and colleagues (in which SP-A was injected into amniotic sacs) might be due to factors specific for these different compartments. Such factors might include soluble elements or differentially functioning resident inflammatory cells. However, in the present study we show that in amniotic fluid macrophages (the cells proposed by Condon to mediate the parturition-inducing effect of SP-A), the effect of SP-A is to suppress the expression of molecules that promote labor. It is also notable that in contrast to the findings of Condon in mice, in human pregnancies fetal macrophages are not observed to cross into maternal tissues [40], [41], and SP-A suppresses the inflammatory cytokine signature of amnion explants [23].

In summary, SP-A suppresses preterm labor and reduces inflammation in response to TLR ligands. This suppression is mediated, at least in part, by TLR2. These observations may hold a key toward preventing preterm birth, which remains the most important preventable cause of neonatal morbidity and mortality in developed countries. Understanding these molecular mechanisms may lead to interventions using SP-A or like molecules to prevent preterm birth.

## Supporting Information

Figure S1 **Immunoidentification using the F4/80 macrophage cell marker.** Adherent cells from peritoneal exudates were exposed to F4/80 or an isotype control antibody, each labeled with the fluorescent dye FITC. Top row: Fluorescent microscopy for FITC stain (green). Bottom row: DAPI nuclear stain (blue), merged with FITC. Original magnification: 200X. Bars: 10 µm.
(TIF)

Figure S2 **The anti-inflammatory effect of SP-A is not mediated through TLR4.** Expression by RT-PCR of IL-1β, CCL5 and TNF-α in WT and TLR4-defective peritoneal macrophages (C3H/HeJ mice) after treatment with PBS, LPS, or PGN+poly(I:C), with or without SP-A. Concentrations of reagents are provided in the text (Methods). P values were calculated by ANOVA and compare four treatment groups (KO or WT with or without SP-A) for each TLR ligand**.** n = 3 replicates per condition per experiment. Depicted is a representative figure from three repeat experiments. Error bars = standard deviation. Values for the PGN+Poly(I:C) group were set to 100.
(TIF)

Figure S3 **Effect of SP-A on phosphorylation of ERK-1 and ERK-2 in macrophages stimulated with TLR ligands.** (A) Western blots showing phosphorylated and total ERK1 and ERK2. Representative blots of 2 independent experiments, each with duplicate sets, are shown. (B) and (C) Densitometric analysis of the ratios of band intensities of phosphorylated to total ERK-1 (B) and ERK-2 (C). P values were calculated by t-test and compare exposures with and without SP-A**.** Error bars = standard deviation.
(TIF)

Figure S4 **Immunoidentification using the F4/80 macrophage cell marker in amniotic fluid macrophages.** Adherent cells from amniotic fluid were exposed to F4/80 or an isotype control antibody, each labeled with the fluorescent dye FITC. Top row: Fluorescent microscopy for FITC (green). Bottom row: DAPI nuclear stain (blue), merged with FITC. Original magnification: 200X. Bars: 10 µm.
(TIF)

Figure S5 **Effect of SP-A on the TLR ligand-induced expression of inflammation markers in the fetal membranes.** RT-PCR analysis of mRNA for IL-1β (A), CCL5 (B), TNF-α (C) and CXCL1 (D) normalized to GAPDH in fetal membrane collected from CD-1 mice on day 14.5 of a 19- to 20-day gestation 8 hours after intrauterine administration of either PBS or PGN+poly(I:C) with or without SP-A. No transcripts were detected for IL-10. Data depicted are expression levels of cytokine for individual mice. N = 7 for control, n = 11 for PGN/poly(I:C), n = 7 for SP-A and n = 12 for SP-A/PGN/poly(I:C). P values were calculated by Mann-Whitney U test and compare PGN+poly(I:C) with and without SP-A.
(TIF)
