# Supplementary figures and images for: Surfactant Protein (SP)-A Suppresses Preterm Delivery and Inflammation via TLR2
Source: PLoS One. 2013 May 20;8(5):e63990. doi: 10.1371/journal.pone.0063990 (PMC3659120; doi:10.1371/journal.pone.0063990)

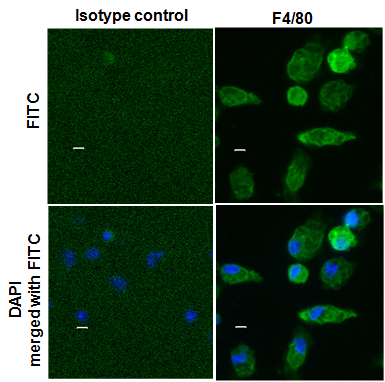

Supplement: Figure S1 — Immunoidentification using the F4/80 macrophage cell marker. Adherent cells from peritoneal exudates were exposed to F4/80 or an isotype control antibody, each labeled with the fluorescent dye FITC. Top row: Fluorescent microscopy for FITC stain (green). Bottom row: DAPI nuclear stain (blue), merged with FITC. Original magnification: 200X. Bars: 10 µm. (TIF) [file pone.0063990.s001.tif]

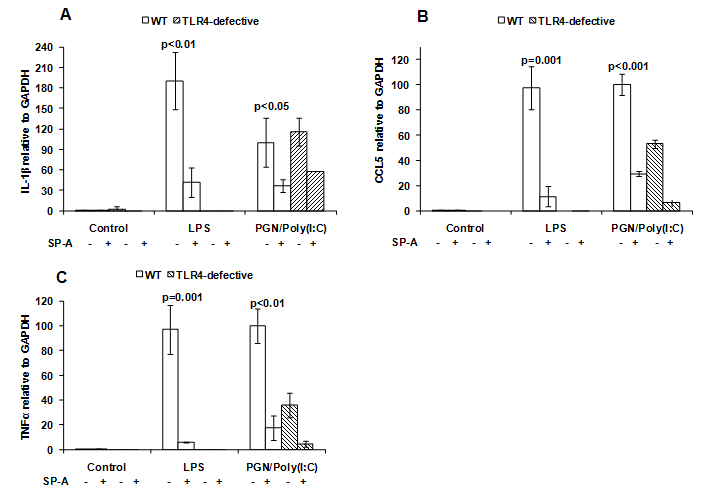

Supplement: Figure S2 — The anti-inflammatory effect of SP-A is not mediated through TLR4. Expression by RT-PCR of IL-1β, CCL5 and TNF-α in WT and TLR4-defective peritoneal macrophages (C3H/HeJ mice) after treatment with PBS, LPS, or PGN+poly(I:C), with or without SP-A. Concentrations of reagents are provided in the text (Methods). P values were calculated by ANOVA and compare four treatment groups (KO or WT with or without SP-A) for each TLR ligand. n = 3 replicates per condition per experiment. Depicted is a representative figure from three repeat experiments. Error bars = standard deviation. Values for the PGN+Poly(I:C) group were set to 100. (TIF) [file pone.0063990.s002.tif]

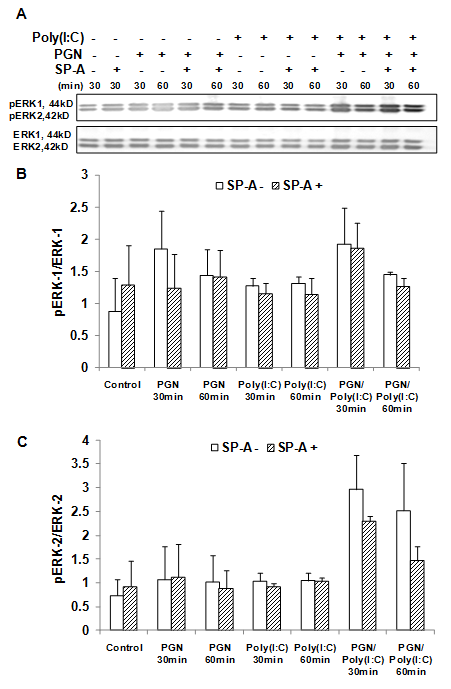

Supplement: Figure S3 — Effect of SP-A on phosphorylation of ERK-1 and ERK-2 in macrophages stimulated with TLR ligands. (A) Western blots showing phosphorylated and total ERK1 and ERK2. Representative blots of 2 independent experiments, each with duplicate sets, are shown. (B) and (C) Densitometric analysis of the ratios of band intensities of phosphorylated to total ERK-1 (B) and ERK-2 (C). P values were calculated by t-test and compare exposures with and without SP-A. Error bars = standard deviation. (TIF) [file pone.0063990.s003.tif]

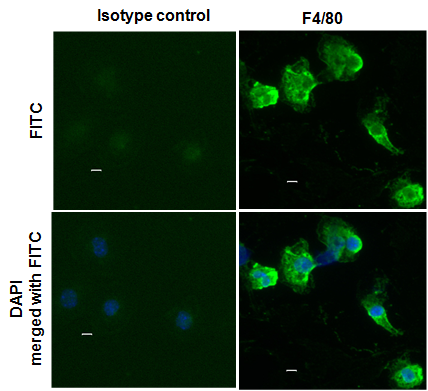

Supplement: Figure S4 — Immunoidentification using the F4/80 macrophage cell marker in amniotic fluid macrophages. Adherent cells from amniotic fluid were exposed to F4/80 or an isotype control antibody, each labeled with the fluorescent dye FITC. Top row: Fluorescent microscopy for FITC (green). Bottom row: DAPI nuclear stain (blue), merged with FITC. Original magnification: 200X. Bars: 10 µm. (TIF) [file pone.0063990.s004.tif]

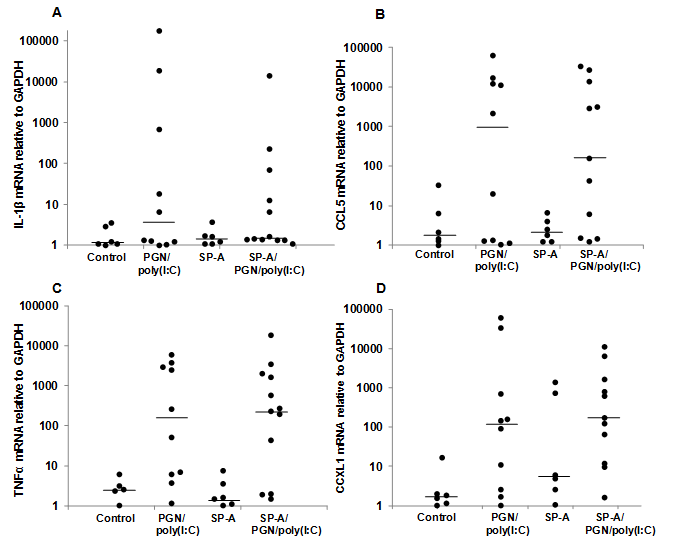

Supplement: Figure S5 — Effect of SP-A on the TLR ligand-induced expression of inflammation markers in the fetal membranes. RT-PCR analysis of mRNA for IL-1β (A), CCL5 (B), TNF-α (C) and CXCL1 (D) normalized to GAPDH in fetal membrane collected from CD-1 mice on day 14.5 of a 19- to 20-day gestation 8 hours after intrauterine administration of either PBS or PGN+poly(I:C) with or without SP-A. No transcripts were detected for IL-10. Data depicted are expression levels of cytokine for individual mice. N = 7 for control, n = 11 for PGN/poly(I:C), n = 7 for SP-A and n = 12 for SP-A/PGN/poly(I:C). P values were calculated by Mann-Whitney U test and compare PGN+poly(I:C) with and without SP-A. (TIF) [file pone.0063990.s005.tif]
